# Family Affluence and the Eating Habits of 11- to 15-Year-Old Czech Adolescents: HBSC 2002 and 2014

**DOI:** 10.3390/ijerph13101034

**Published:** 2016-10-24

**Authors:** Jaroslava Voráčová, Erik Sigmund, Dagmar Sigmundová, Michal Kalman

**Affiliations:** Faculty of Physical Culture, Institute of Active Lifestyle, Palacký University Olomouc, Tr. Miru 117, Olomouc 77111, Czech Republic; erik.sigmund@upol.cz (E.S.); dagmar.sigmundova@upol.cz (D.S.); michal.kalman@upol.cz (M.K.)

**Keywords:** eating habits, children, adolescents, HBSC, FAS, socioeconomic status, Czech Republic

## Abstract

Socioeconomic inequalities in eating habits have a profound impact on the health of adolescents. The aim of the present study was to evaluate socioeconomic disparities in the eating habits of Czech adolescents and to compare their change between 2002 and 2014. The data from the Czech Health Behavior in School-aged Children (HBSC) study conducted in 2002 and 2014 was utilized. The Family Affluence Scale (FAS) was used to assess socioeconomic disparities. Higher odds of daily consumption of fruit (2002: OR = 1.67; 2014: OR = 1.70, *p* < 0.001) and vegetables (2002: OR = 1.54; 2014: OR = 1.48, *p* < 0.001) were associated with high FAS in both genders. Adolescents with higher FAS were less likely to consume sweets (2002: OR = 0.72, *p* < 0.05) and more likely to eat breakfast on weekdays (2014: OR = 1.19, *p* < 0.05). In 2002 and 2014, the data showed lower odds of daily consumption of soft drinks (Low: OR = 0.47; Medium: OR = 0.43; High: OR = 0.41, *p* < 0.001), fruit (Low: OR = 0.73; Medium: OR = 0.74, *p* < 0.001; High: OR = 0.75, *p* < 0.05), sweets (Low: OR = 0.71; Medium: OR = 0.79, *p* < 0.001) and breakfast on weekends (High: OR = 0.70, *p* < 0.05), and a higher likelihood of eating breakfast on weekdays (Low: OR = 1.26, *p* < 0.01; Medium: OR = 1.13, *p* < 0.05). These findings play an important role in future public measures to improve dietary habits and decrease social inequalities in youth.

## 1. Introduction

Socioeconomic inequalities in eating behaviors are linked to overweight and obesity and have a profound impact on health and well-being [1,2,3,4,5]. Most recent studies representing data from countries with various levels of income have shown that children and adolescents from families with low socioeconomic status (SES) tend to have poorer diets [1,6,7,8], high levels of sedentary activity [8,9], higher likelihood of smoking cigarettes [8], higher odds of obesity [9,10,11,12] and an increased risk of morbidity and mortality [13]. Unhealthy diets consisting of sugar-rich and fatty foods among children are also common in families with low parental education levels [1,14]. Healthy foods are frequently more expensive and are not often affordable for families with lower incomes [15,16], which may widen disparities in access to healthy diets [15].

Differences in eating patterns with regard to SES and gender can be seen from young ages [14]. Several studies demonstrated that SES plays one of the key roles in the development of healthy eating habits in children [1,17], which tend to be carried into adulthood [18,19,20]. Significant associations have been documented between parental SES and the consumption of breakfast [7,9,21,22], a morning snack [9], dinner [21], fruit and vegetables [7,21,23,24,25] and dairy products (low and full-fat milk/yoghurt, chocolate milk, low and full-fat cheese and feta cheese) [7,25] in children and adolescents. Sweet beverages, processed and energy-dense foods have been inversely associated with family SES [1,6]. These findings suggest a higher risk of developing overweight and obesity during childhood in low-income families [9,26], and therefore there is a need to address the socioeconomic disparities by developing policies and programs that will improve the eating habits of children from these social groups.

Over the last two decades, international studies have adopted the Family Affluence Scale (FAS) as a measurement of parental SES [27]. The FAS, comprising easily answered questions that reflect material affluence, has proven to be a useful indicator of child material affluence [27]. Several studies, using the data from the Lithuanian, Scottish and Norwegian Health Behavior in School-aged Children (HBSC) questionnaire and the FAS, monitored the relationship between family material wealth and dietary frequencies (consumption of fruit, vegetables, sweets and/or sugar-sweetened beverages) and examined time trends in socioeconomic differences in these eating behaviors [28,29,30]. The current findings show that in spite of some improvements in dietary trends across all SES levels, the persistent socioeconomic inequalities in eating habits still exist and need to be addressed in future programs and policies [28,29].

In the Czech Republic, dietary trends in children and adolescents have been monitored and documented over the last 12 years [31] in addition to trends in overweight/obesity, physical activity and screen time [32]. However, while socioeconomic disparities in dietary behaviors are well documented across Europe, little is known about the association between eating habits and FAS/SES and its changes over time in Czech children and adolescents. This information is important for identifying social groups who may be at higher risk and for developing more effective public health interventions and policies aiming to improve children’s diets. Therefore, the aims of this study were (i) to determine the association between the dietary habits of Czech adolescents and family socio-economic position and (ii) to examine the change in socioeconomic disparities in six eating behaviors between 2002 and 2014.

## 2. Materials and Methods

### 2.1. Study Design

This study used data from the Czech HBSC study conducted in 2002 and 2014. The HBSC is a cross-national survey undertaken in collaboration with the World Health Organization (WHO) every four years in member countries [33]. This study followed the standardized protocol developed by the HBSC International Network committee (No. 17/2013), which includes detailed information on methodology, conceptual framework, survey design and administration, and translation guidelines [33]. The External Protocol for the HBSC 2014 survey is available to the public on the HBSC website [34].

For each year of data collection, the HBSC questionnaire contains mandatory (demographic factors, social context, health outcomes, health and risk behaviors), optional and country-specific questions that assess adolescents’ health, well-being and health behaviors in the social context. In this study, only data pertaining to eating behaviors and family affluence were used. Participants were 11-, 13- and 15-year-old children and adolescents who were selected by stratified cluster sampling. The data were collected through self-completed questionnaires administered in the classroom. After adjustment for missing data, the file was exported to the HBSC International Data Bank at the University of Bergen to be compiled within an international data file. The outcome variables were dichotomized. In both years the response rates were between 75% and 85%.

### 2.2. Family Socioeconomic Status (SES)

Assessing SES by using income, education or occupation in young people results in high levels of missing data [27]; therefore, this study used the FAS as a simple indicator of affluence in the children’s home [35]. The FAS consisted of several easily answered questions designed to quantify material assets in the family [27,35]. Because of the fast changes in economic circumstances and common material assets in the families, the FAS questions have to be adapted to remain discriminatory within very high or poor affluent countries [35,36]. High validity (kappa coefficients 0.41%–0.74%; 76.2%–88.1% agreement) and moderate reliability (Cronbach’s α = 0.58) between children and parental responses on the FAS items have been documented [37,38,39,40].

### 2.3. Family Affluence Scale (FAS)

In 2002, the FAS was composed of four items: (1) Car: does your family own a car, van or truck? (Codes: No = 0; One = 1; Two or more = 3); (2) Own bedroom: do you have your own bedroom for yourself? (Codes: No = 0; Yes = 1); (3) Holidays: during the past 12 months, how many times did you travel away on holiday with your family? (Codes: Never = 0; Once = 1; Twice = 2; Three or more times = 3); and (4) Computers: how many computers does your family own? (Codes: None = 0; One = 1; Two = 2; Three or more = 3) [27]. Family affluence was calculated by the summation of answers into a scale from 0 to 9, and participants were divided into tertiles (affluence: low = 0–3, medium = 4–6, high = 7–9) [38]. In 2014, the updated version of the FAS was used to compensate for the changing social environment [35,36]. Two new questions were added to the existing items on having a bedroom, car, family holidays and computer ownership: (1) Does your family have a dishwasher? (Codes: No = 0; Yes = 2); and (2) How many bathrooms (room with a bath/shower or both) are in your home? (None = 0; One = 1; Two = 2; Three or more = 3). Answers were ranked on a scale from 0 to 13 and then categorized as low (0–6), medium (7–9) and high (10–13) family affluence [35].

### 2.4. Eating Habits

The frequency of six eating habits was assessed by questions: “How many times a week do you consume fruit/vegetables/sweetened soft drinks/sweets?” (Response options: never/less than once a week/two to four times a week/five to six times a week/once a day/more than once a day) and “How often do you usually have breakfast (more than a glass of milk or fruit juice)?” (Response options: weekdays: never/one day/two days/three days/four days/five days; weekends: never/only on one day/on both days). Daily consumption referred to eating fruit/vegetables/soft drinks/sweets once or more per day and eating breakfast every day during weekdays and on both days on the weekends.

### 2.5. Statistical Analyses

Frequencies of the consumption of eating habits were calculated for each gender and survey year (2002 and 2014) (Table 1). A possible interaction of the daily consumption of eating habits with family affluence for each gender was assessed using logistic regression (Enter method). Logistic regression was also used to calculate changes in eating habits for each gender and family affluence group between 2002 and 2014. The likelihood of daily consumption was described by odds ratios (ORs) with 95% confidence intervals (CIs). The reference group for family affluence was low and for time changes the reference year was 2002. Statistical analyses were performed on IBM SPSS v21.0 software (IBM SPSS, Inc., Chicago, IL, USA).

## 3. Results

The total number of participants who completed the HBSC questionnaire was 5012 (2412 boys; 2600 girls) in 2002 and 5819 (2843 boys; 2976 girls) in 2014. Table 1 describes the frequency of consumption of fruit, vegetables, sweetened soft drinks, sweets and breakfast (weekdays and weekends) according to the FAS category in 2002 and 2014, as well as the changes in socioeconomic inequalities in the eating habits of Czech adolescents over time.

### 3.1. Overall Socioeconomic Inequalities

When comparing low FAS levels to medium and high FAS levels, children from more affluent families were significantly (*p* < 0.001) more likely to consume daily fruit and vegetables in both 2002 and 2014 (Table 1). Substantial social inequalities were also found in the regular consumption of sweets in 2002 (low vs. high FAS, *p* < 0.05) and breakfast during weekdays in 2002 (low vs. medium FAS, *p* < 0.01) and 2014 (low vs. high FAS, *p* < 0.05). No differences between the FAS groups were found for soft drink and breakfast (weekends) intake in both years, as well as daily sweets in 2014.

### 3.2. Inequalities in Family Affluence by Gender

In 2002, significantly higher odds of daily consumption of fruit and vegetables were associated with medium (OR = 1.29/1.36 in boys/girls; OR = 1.28/1.33 in boys/girls, respectively) and high (OR = 1.64/2.05 in boys/girls; OR = 1.94 in girls only, respectively) FAS in both genders. Similar findings were indicated in 2014, when adolescents from medium (OR = 1.37 in girls only; OR = 1.32/1.30 in boys/girls, respectively) and high (OR = 1.55/1.91 in boys/girls; OR = 1.44/1.59 in boys/girls, respectively) affluence families had a greater likelihood of eating fruits and vegetables compared to adolescents from lower affluence families. In both genders, FAS was not significantly linked to the daily consumption of sweetened soft drinks (2002 and 2014), sweets (2014) and breakfast on weekdays (2014). In addition, no associations between the FAS were found with the daily consumption of sweets and breakfast during weekdays in boys (2002) and regular breakfast on the weekend in girls (2002) and boys (2014). Greater disparities in family material wealth between the FAS groups were seen in the daily consumption of sweets (girls), breakfast during weekdays (girls) and on weekends (boys) in 2002 compared to 2014 (Table 1).

### 3.3. Changes in Eating Habits by Family Affluence between 2002 and 2014

From 2002 to 2014, the data showed a significant (*p* < 0.001) decrease in the percentages of daily consumption of sweetened soft drinks (both genders) and fruit (girls only) across all FAS groups (Table 1). Additionally, a significant improvement was indicated in the low and medium FAS groups regarding the regular consumption of sweets (both genders) and breakfast during weekdays (girls only). Daily vegetable intake (both genders) and breakfast during weekdays (boys only) and on weekends (both genders, except girls from low FAS and boys from high FAS) remained statistically unchanged in all FAS groups over time.

## 4. Discussion

This study examined the association between eating habits and FAS levels in Czech adolescents as well as monitoring the changes in socioeconomic disparities in eating habits from 2002 to 2014. Substantial inequalities in the daily consumption of fruit and vegetables were found to be associated with low FAS in the 2002 and 2014 surveys. Moreover, greater disparities among the FAS groups were seen in the intake of sweets (girls), breakfast during weekdays (girls) and on the weekends (boys) in 2002. No differences between FAS groups were found in the daily intake of sweetened soft drinks, sweets and breakfast during weekdays in both genders in 2014. Over the 12-year period, significant improvements were seen in the daily consumption of sweetened soft drinks (all FAS groups, both genders), sweets (low and medium FAS, both genders), and breakfast during the weekdays (low and medium FAS groups, girls) and on the weekends (low FAS, girls, and high FAS, boys). Our findings also showed a reduction in fruit intake in children from families with low and medium FAS, and there was no change in vegetable consumption.

The FAS is a valid instrument for measuring material wealth [37,38] and is often used to provide information on parental SES [27]. Several cross-sectional and longitudinal studies have documented the associations between diet and SES (measured by parental income and/or education) in adolescents [8,14,17,41,42,43]. The results from a systematic review addressing this relationship indicated that 88% of high quality studies reported that low SES adolescents have a poorer diet (greater fat and refined sugar intake, lower amounts of protein, monounsaturated fat and most vitamins and minerals) compared with their peers from high SES families [8,44]. Similar to our findings, a negative association between fruit and vegetable consumption in youth and family SES has been reported in previous studies across Europe [1,7,8,17,23,28]. Although social inequalities in the diets of children and adolescents still persist, a study of Nordic adolescents showed no differences in fruit and vegetable intake between FAS groups or gender as a result of public measures implemented over the past decade [41,43]. According to the current literature, lower family affluence is also associated with lower daily breakfast intake in most countries [7,28,45] and a higher intake of soft drinks with sugar [17,41,46], which is in contrast with our findings. In the Czech Republic there are currently no programs targeted at improving socioeconomic disparities in adolescents. No change in consumption of soft drinks and breakfast across FAS levels might be explained by various strategies that promote healthy eating [47,48,49,50,51].

Eating habits in adolescents are attributable to multiple factors such as food availability at home, food preferences, cost, convenience, school support, personal and cultural beliefs and parental modeling and permissiveness [52,53,54]. Socioeconomic disparities in the availability of healthy foods may be influenced by parents’ knowledge of nutrition [41] and food cost [16,55]. The literature has shown that parents with higher education and income levels have better knowledge of dietary guidelines and therefore have a greater positive impact on their children’s diets compared to parents of lower SES [17,41]. The cost and affordability of a healthy diet may be another barrier to healthy eating for people with low incomes [16,55]. A study by Barosh [55] demonstrated that low-income households had to spend up to 48% of their weekly income to buy healthy and sustainable food compared to 9% of the weekly income of high-income families [55]. In addition, unhealthy diets in people with low SES might be influenced by taste preferences for less healthy food items [56] that were mediated by the repeated introduction of unhealthy foods at an early age, which affected children’s taste acquisition and eating habits later in life [57]. Young children tend to refuse unfamiliar foods eight to 15 times before accepting them and, therefore, future eating habits can be influence by repeated provision of a wide variety of food items in early childhood [57]. Children’s intake and preferences are also influenced by eating habits of their parents [58]. According to a study of eating behavior, high-income families are more likely to repeatedly introduce healthy foods, regardless of high cost, that their children initially refuse compared to the families with low incomes [57]. This might explain the disparities in vegetable and fruit consumption between FAS levels.

Research on the socioeconomic inequalities in the eating habits of Czech children and adolescents is limited. To our knowledge, the HBSC study is the only study that monitored the relationship between family affluence and eating habits in young people [35,45,59,60]. In the Czech Republic, few previous studies have examined social disparities among adults [61,62,63] and children/adolescents [45,64,65], but only one study researched eating habits (daily breakfast consumption, the HBSC study) [45]. The limited number of studies addressing inequalities in eating habits may result in the lack of public measures that focus on decreasing social disparities among Czech young people.

Even though there are some programs addressing healthy eating in children and adolescents in the Czech Republic, most of these programs are still in the testing/trial phase and many do not apply to the entire youth population [47,48,49,50,51]. For example, the low effectiveness of the “free fruit (renamed to fruit and vegetables) at school program” (which had a low budget allocation, and provided one piece of fruit or vegetable only twice a month) could explain the persistent socioeconomic inequalities in fruit and vegetable consumption in Czech youth, as well as a significant decrease in fruit intake across all FAS groups in girls and in boys in medium FAS groups, and no change in daily vegetable consumption from 2002 to 2014. Therefore, our findings suggest a need for developing and implementing initiatives and programs that will focus on the promotion of vegetable (both genders) and fruit (especially in boys) consumption, targeting children from less affluent families.

### Strengths and Limitations

The main strengths of this study are the large sample size, which is representative of the Czech adolescent population aged 11–15, the standardized procedures of the international cross-sectional HBSC study, the high response rates and the use of the FAS as an effective measure of material wealth with minimum missing data.

However, several limitations should be taken into consideration. Reported eating habits may have been affected by social desirability and approval bias, leading to an overestimation of the frequency of the consumption of healthy foods and an underestimation of the consumption of unhealthy foods [66,67,68]. Evidence suggests that people with higher education tend to over-report the consumption of healthy food items more frequently [69] which could decrease the gap between low and high FAS groups in terms of daily consumption of fruit and vegetables. Biased reporting is another disadvantage that may include misinterpreting or misunderstanding of the questions, lack of motivation and not paying attention while filling out the questionnaire. A study on European adolescents found that weight status and psychosocial weight-related factors are the major correlates of misreporting of dietary energy [70]. Also, the questions used to measure eating habits were limited to information on the consumption of five food items (fruit, vegetables, soft drinks, sweets and breakfast) and did not measure the quantities of food eaten and whether children met the dietary recommendation. Additionally, this study had a cross-sectional design, and causality cannot be established from our results. Therefore, longitudinal studies are needed in order to gain more information on the direction of the relationship between SES (or FAS) and eating habits.

Moreover, the assessment of family SES by the FAS should be taken into account when interpreting the results. The FAS is a tool that measures more material wealth than SES among young people because it does not include information on parental education, income and/or occupation. Validation studies found that the FAS is a valid instrument for measuring family affluence [38,71]. In addition, caution is encouraged when comparing the FAS between different times, as some FAS items may be measured differently in different survey years [71]. For example, owning a computer in 2002 was related to a game culture, whereas in 2014 computers were used for educational purposes [71]. Additionally, in our study, the FAS was calculated in a different way (questionnaire items) in 2002 and 2014. It was adapted to changes in economic circumstances so the questions remained discriminatory between low and high affluent populations.

## 5. Conclusions

The aim of this study was to provide information on the socioeconomic inequalities in six eating habits of Czech children and adolescents and to monitor their change over time. Substantial social disparities were found in the consumption of fruit and vegetables in both genders and both survey years. Significant differences were also observed between low and high FAS in the intake of sweets (2002) and breakfast during the weekdays (2014). These findings suggest that the socioeconomic inequalities have not changed much from 2002 to 2014. In 2002 and 2014, the odds of the daily consumption of soft drinks (all FAS groups), fruits (all FAS groups), sweets (low and medium FAS) and breakfast on the weekends (high FAS) decreased, while the odds of eating breakfast during weekdays (low and medium FAS) increased. These results contribute to a better understanding of the current situation in the Czech Republic and suggest that future public measures to improve healthy eating among Czech young people should focus on decreasing gender and social inequalities in eating habits (especially with respect to fruit and vegetable consumption) by creating intervention programs that target less affluent families.

## Figures and Tables

**Table 1:** Odds ratios of daily consumption by family affluence, 2014 vs. 2002.

FAS 2002								FAS 2014							2014 vs. 2002					
OR ^1–6^	Low	Medium	High	Low vs. Medium		Low vs. High		Low	Medium	High	Low vs. Medium		Low vs. High		Low FAS		Medium FAS		High FAS	
	% ^a^	% ^a^	% ^a^	OR	95% CI	OR	95% CI	% ^a^	% ^a^	% ^a^	OR	95% CI	OR	95% CI	OR	95% CI	OR	95% CI	OR	95% CI
**Daily soft drinks ^1^**	28.9	28.6	29.6	1.00	(0.87–1.14)	1.08	(0.85–1.37)	16.0	14.8	15.0	0.91	(0.76–1.10)	0.94	(0.75–1.17)	0.47 ***	(0.39–0.56)	0.43 ***	(0.37–0.50)	0.41 ***	(0.31–0.53)
Boys	30.8	30.8	34.2	1.00	(0.83–1.21)	1.17	(0.85–1.61)	16.6	16.9	16.7	1.02	(0.78–1.34)	1.01	(0.74–1.38)	0.45 ***	(0.34–0.59)	0.46 ***	(0.38–0.55)	0.39 ***	(0.27–0.55)
Girls	27.0	26.3	25.0	0.97	(0.81–1.16)	0.90	(0.62–1.31)	15.3	12.7	13.2	0.81	(0.62–1.05)	0.84	(0.62–1.15)	0.49 ***	(0.38–0.63)	0.41 ***	(0.33–0.50)	0.46 ***	(0.30–0.70)
**Daily sweets ^2^**	26.9	24.6	20.3	0.90	(0.78–1.02)	0.72 *	(0.55–0.94)	20.7	20.5	22.8	0.99	(0.84–1.17)	1.13	(0.94–1.37)	0.71 ***	(0.60–0.84)	0.79 ***	(0.69–0.90)	1.12	(0.84–1.48)
Boys	26.9	24.6	25.1	0.89	(0.73–1.08)	0.91	(0.65–1.28)	21.1	20.0	23.5	0.93	(0.72–1.19)	1.14	(0.87–1.50)	0.73 *	(0.57–0.94)	0.77 **	(0.63–0.93)	0.92	(0.64–1.31)
Girls	26.8	24.6	15.5	0.89	(0.74–1.07)	0.50 **	(0.32–0.78)	20.2	20.9	22.1	1.04	(0.83–1.31)	1.12	(0.86–1.46)	0.69 **	(0.55–0.87)	0.81 *	(0.67–0.97)	1.55	(0.98–2.46)
**Daily fruit ^3^**	37.5	44.2	52.0	1.28 ***	(1.14–1.45)	1.67 ***	(1.34–2.08)	30.9	36.8	43.5	1.30 ***	(1.12–1.50)	1.70 ***	(1.44–2.00)	0.73 ***	(0.63–0.85)	0.74 ***	(0.66–0.83)	0.75 *	(0.59–0.94)
Boys	31.3	37.0	42.7	1.29 **	(1.07–1.55)	1.64 **	(1.20–2.22)	28.0	32.5	37.7	1.24	(0.99–1.55)	1.55 ***	(1.22–1.99)	0.86	(0.68–1.08)	0.82 *	(0.69–0.97)	0.81	(0.59–1.11)
Girls	43.7	51.3	61.3	1.36 ***	(1.16–1.60)	2.05 ***	(1.47–2.86)	33.8	41.1	49.3	1.37 ***	(1.13–1.66)	1.91 ***	(1.53–2.39)	0.66 ***	(0.54–0.80)	0.66 ***	(0.57–0.77)	0.62 **	(0.43–0.87)
**Daily vegetables ^4^**	24.1	29.3	34.4	1.28 ***	(1.12–1.6)	1.54 ***	(1.22–2.00)	22.9	27.9	30.9	1.29 ***	(1.10–1.52)	1.48 ***	(1.24–1.77)	0.93	(0.79–1.00)	0.94	(0.83–1.07)	0.89	(0.70–1.14)
Boys	20.7	25.1	26.5	1.28 *	(1.04–1.58)	1.38	(0.98–1.94)	19.7	24.4	26.1	1.32 *	(1.03–1.69)	1.44 **	(1.09–1.90)	0.94	(0.72–1.23)	0.97	(0.80–1.16)	0.98	(0.69–1.39)
Girls	27.4	33.4	42.3	1.33 **	(1.11–1.58)	1.94 ***	(1.39–2.71)	26.0	31.3	35.7	1.30 *	(1.06–1.60)	1.59 ***	(1.25–2.01)	0.93	(0.75–1.16)	0.91	(0.77-1.07)	0.76	(0.54–1.08)
**Daily breakfast (weekday) ^5^**	49.4	53.5	53.1	1.23 **	(1.09–1.38)	1.24	(0.99–1.54)	54.2	56.6	58.3	1.11	(0.96–1.27)	1.19 *	(1.01–1.40)	1.26 **	(1.09–1.45)	1.13 *	(1.01–1.27)	1.21	(0.96–1.53
Boys	57.5	59.2	57.3	1.07	(0.89–1.28)	0.99	(0.73–1.34)	55.9	59.0	60.8	1.14	(0.93–1.40)	1.22	(0.97–1.55)	0.93	(0.75–1.16)	0.99	(0.84–1.17)	1.15	(0.84–1.58)
Girls	41.2	47.8	48.8	1.31 **	(1.11–1.54)	1.36	(0.98–1.89)	52.5	54.2	55.8	1.07	(0.89–1.29)	1.14	(0.92–1.43)	1.58 ***	(1.30–1.92)	1.29 ***	(1.11–1.51)	1.33	(0.94–1.87)
**Daily breakfast (weekend) ^6^**	84.9	85.6	88.4	1.04	(0.88–1.23)	1.37	(0.97–1.92)	83.2	85.1	84.5	1.18	(0.98–1.42)	1.10	(0.89–1.37)	0.87	(0.71–1.06)	0.99	(0.84–1.15)	0.70*	(0.49–0.99)
Boys	83.0	84.8	90.4	1.14	(0.90–1.44)	1.93 **	(1.19–3.14)	83.2	82.1	82.1	0.93	(0.70–1.22)	0.92	(0.68–1.26)	1.02	(0.76–1.36)	0.83	(0.67–1.03)	0.49 **	(0.30–0.80)
Girls	86.7	86.3	86.3	0.97	(0.76–1.22)	0.97	(0.60–1.56)	83.1	88.1	86.8	1.51 **	(1.16–1.97)	1.34	(0.98–1.82)	0.76 *	(0.58–1.00)	1.18	(0.94–1.49)	1.04	(0.63–1.72)

Note: FAS—family affluence scale; % ^a^—percentage of adolescents who performed the eating behaviour at least daily; OR ^1–6^ of daily consumption for each behaviour; logistic regression Enter method (LR): low vs. medium/low vs. high FAS group (reference group is low FAS)/2014 vs. 2002 for each FAS group (reference group is a cohort of 2002: OR—odds ratio, 95% CI—95% confidence interval, * *p* < 0.05, ** *p* < 0.01, *** *p* < 0.001.
